# Synthesis of Binderless ZK-4 Zeolite Microspheres at High Temperature

**DOI:** 10.3390/molecules23102647

**Published:** 2018-10-16

**Authors:** Elyssa G. Fawaz, Darine A. Salam, Habiba Nouali, Irena Deroche, Severinne Rigolet, Benedicte Lebeau, T. Jean Daou

**Affiliations:** 1 Université de Haute Alsace (UHA), CNRS, Axe Matériaux à Porosité Contrôlée (MPC), Institut de Science des Matériaux de Mulhouse (IS2M), UMR 7361, 3 bis rue Alfred Werner, F-68093 Mulhouse, France; egf00@mail.aub.edu (E.G.F.); habiba.nouali@uha.fr (H.N.); irena.deroche@uha.fr (I.D.); severinne.rigolet@uha.fr (S.R.); benedicte.lebeau@uha.fr (B.L.); 2 Université de Strasbourg (UniStra), F-67081 Strasbourg, France; 3 Department of Civil and Environmental Engineering, Faculty of Engineering and Architecture, American University of Beirut, P.O. Box 11-0236, Riad El Solh, Beirut 1107-2020, Lebanon; ds40@aub.edu.lb

**Keywords:** zeolites, ZK-4 zeolite, microspheres, molecular decontamination, volatile organic compounds (VOCs)

## Abstract

Binderless zeolite macrostructures in the form of ZK-4 microspheres were prepared using anion-exchange resin beads as shape-directing macrotemplates. The particles were synthesized under hydrothermal conditions at different temperatures and treatment times. The influence of the different synthesis parameters was investigated by X-ray diffraction, scanning electron microscopy, fluorescence X, nitrogen adsorption measurements and ^29^Si solid-state NMR. Fully crystalline spheres similar in size and shape to the original resin beads were obtained by a hydrothermal treatment at the highest temperatures (150–180 °C) for a short treatment time of 24 h. The synthesized microspheres showed to be promising in the molecular decontamination of volatile organic compounds (VOCs).

## 1. Introduction

Because of their complex microporous structure, zeolites have a very high specific surface area. This makes them effective at adsorbing a wide variety of substances. The adsorption properties of zeolites vary with their porous structure and affinity to different molecules [1,2,3,4]. Synthetic aluminosilicate zeolites are among the most widely produced zeolites and used in many applications in research and industry [5,6,7,8]. However, commercialized powder zeolites often present a low molecular sieve performance limiting the mass transfer of reactant and product molecules and causing deactivation of adsorbents [9,10,11,12,13,14]. A secondary dust contamination due to particles breeding could also occur [12,13,14]. 

In order to overcome these constraints, zeolite crystal size reduction (<100 nm) through nanozeolite preparation [15], and assembly of nanozeolites into hierarchical porous materials by introducing secondary larger porosity into the zeolite’s intrinsic microporous framework, have been proposed [16,17,18,19,20]. Also, binding additives have been used as post-synthetic modification agents to convert the zeolite powder into pellets, tablets, films or other shapes suitable for practical use [10,11]. However, binding additives introduced in amounts of up to 50% (*w*/*w*) affect the zeolite’s adsorption properties and block its pores. Thus, the possibility of tailoring molecular sieve macrostructures without the addition of binders, while addressing process restrictions such as diffusion limitations, is required. 

Therefore, self-bonded nanozeolite spherical macrostructures prepared directly or through templating [21] have attracted special attention for their ease of manipulation and substantial advantages such as high hydrothermal stability, improved diffusion of reactants, reduced diffusion path length, and postponed catalyst and sorbent deactivation [22,23]. Efforts have been dedicated to the synthesis of hierarchical zeolites, such as the assembly of zeolite nanocrystals [24,25,26], use of soft templates [27,28,29,30], and confined synthesis of zeolites in templates [15,31,32]. However, the control of the crystal morphology and secondary mesoporous structure has been challenging. 

Zeolite microspheres with hierarchical pores are emerging as attractive controlled and stable materials for applications in adsorption and catalysis [33,34]. Hollow zeolite spheres have been fabricated by assembly of nanozeolites into macroscopic structures and removal of templates. Yue et al. [35] synthesized hollow zeolite spheres of silicalite-1 using oil/water emulsions as templates whereby the oil phase acts as a template in the hydroformation of the microspheres. Chen et al. [36] produced silicalite-1 hollow spheres that exhibit lamellar nanoshell morphology by involving the integrated use of soft (multiquaternary ammonium surfactants) and hard templates (macroporous carbon). Pashkova et al. [37] synthesized hollow spheres of ZSM-5 zeolite crystals, requiring neither a hard/soft template for sphere formation nor a structure-directing agent. In this case, the same aluminosilicate precursor was used as a source of silica and alumina for zeolite crystallization, and played the role of a shape-directing agent for the formation of hollow microspheres. Yan et al. [38] prepared hollow zeolite NaA/chitosan composite microspheres using premodified chitosan solution-coated calcium alginate microspheres that served as template of the hollow structure. 

Aside from hollow spheres, zeolite microspheres composed of small crystals were prepared by using templates, which, upon removal, determine the pore structure of the products. Wang et al. [39] developed a new method for the preparation of silicalite-1 microspheres using impregnated monodispersed micron-sized poly-styrene-*co*-divinylbenzene porous particles as template. Yin et al. [26] used dimethyldiallyl ammonium chloride acrylamide copolymer as a template for the fast and one-step formation of nanozeolite beta microspheres. Tao et al. [32] and Yang et al. [20] reported a space-confined synthesis route of hierarchical MFI and beta zeolite microspheres with nanorod-oriented assembled structures of a carbon–silica composite monolith via hydrothermal treatment. Wang et al. [40] hydrothermally synthesized hierarchical ZSM-5 zeolite microspheres by using organofunctionalized silanized mesoporous silica as silica source. Sashkina et al. [41] reported the synthesis of aerogel/zeolite composite microspheres based on Fe-containing zeolite nanocrystals embedded into silica aerogel matrix using the emulsion/gelation technique. The technique entails varying the stirring rate during emulsification to determine the size and texture of the composite microspheres. 

The reported methods have the disadvantage of using expensive templates. Therefore, simple and low-cost templates to synthesize zeolite objects with mesoporosity, such as anion-exchange resins, are needed. The preparation of spherical macrostructures employing anion-exchange resin beads as templates was reported previously by Tosheva et al. [21,42] and Yin et al. [43] for MFI and *BEA-type zeolites, and MFI-type zeolite, respectively. To our knowledge, no studies had reported the one-shot synthesis of zeolite beads composed of small ZK-4 crystals with hierarchical porosity involving intercrystalline mesopores. This work will therefore address the production of ZK-4 zeolite microspheres using ion-exchange resins as shape-directing macrotemplates. The adsorption efficiency of the produced ZK-4 molecular sieves will be tested in the molecular decontamination of volatile organic compounds (VOCs). The study will evaluate the effect of temperature and treatment time on the synthesis of pure and well-crystallized macrostructures of zeolites, and assess their ability to meet VOC adsorption requirements. 

## 2. Methodology

### 2.1. Preparation of the ZK-4 Zeolite Microspheres 

For the zeolite synthesis, a solution was prepared by mixing corresponding amounts of silica colloidal solution (Sigma-Aldrich (Saint Louis, MO, USA), LUDOX HS 30 wt % SiO_2_ in water), aluminum isopropylate (Sigma-Aldrich), sodium hydroxide (NaOH), and pentahydrated tetramethylammonium hydroxide (TMAOH, 5 H_2_O) (Sigma-Aldrich). Alumina solution was prepared by dissolving the aluminum isopropylate in an aqueous solution containing NaOH and TMAOH. The LUDOX HS was then added to the alumina solution under intensive stirring to obtain a clear solution. This clear homogeneous solution with the molar composition 14(TMA)_2_O:0.8Na_2_O:11.9SiO_2_:700H_2_O:1.9Al_2_O_3_ was transferred into 48 mL PTFE-lined stainless steel autoclaves (Top Industrie, Vaux-le-Pénil, France) together with the macroporous strongly basic styrene–divinylbenzene anion-exchange resin beads (Dowex MSA-1) which were added to the synthesis solution in a weight ratio of 20:1. The autoclaves were then heated at different temperatures ranging from 70 to 180 °C for treatment time ranging from 24 h to 7 days. The MSA-1 beads were used as shape-directing macrotemplates to produce zeolite ZK-4 spheres. 

After hydrothermal synthesis, the resin–zeolite ZK-4 composites were separated from the mother liquor and the zeolite crystallized in the bulk, treated in a 0.1 M ammonia solution in an ultrasonic bath for 5 min, rinsed several times by suspension in distilled water, and lastly, decanted and dried at 60 °C overnight. Finally, the organic macrotemplate and the tetramethylammonium cations (TMA^+^) occluded in the zeolite pores were removed by calcination at 550 °C at a rate of 1 °C/min for a duration of 5 h.

### 2.2. Characterization of Zeosils

The purity and the crystallinity of the calcined zeolite microspheres were checked by XRD analysis (MPD X’Pert Pro, PANalytical, Paris, France). X-ray diffraction patterns of the different samples were recorded using a PANalytical MPD X’Pert Pro diffractometer operating with Cu Kα radiation (λ = 0.15418 nm) equipped with an X’Celerator real-time multiple strip detector (active length = 2.122° 2θ). The powder pattern was collected at 22 °C in the range 3 < 2θ < 50° with a 2θ angle step of 0.017° and a time step of 220 s. The unit cell parameters were refined using the Win XPow software (version 2.20, STOE, Darmstadt, Germany). The size and the morphology of the calcined zeolite microspheres were determined by scanning electron microscopy (SEM) using a Philips XL 30 FEG microscope (Tokyo, Japan).

Nitrogen adsorption/desorption isotherms were measured using a Micromeritics ASAP 2420 apparatus (Norcross, GA, USA). Prior to the adsorption measurements, the calcined samples were outgassed at 300 °C overnight under vacuum. The specific surface area (S_BET_) and microporous volume (V_micro_) were calculated using the BET and *t*-plot methods, respectively. Mesoporous volume was found by subtracting the microporous volume from the total porous volume. 

The Si/Al molar ratio of the microspheres was estimated using two different methods including X-ray fluorescence (Philips, Magic X, Tokyo, Japan) and solid-state NMR spectroscopy. 

^29^Si (I = 1/2) magic angle spinning (MAS) NMR spectra were recorded at room temperature, with a Bruker Avance II 300 Mhz spectrometer operating at B_0_ = 7.2 T (Larmor frequency ν_0_ = 59.62 MHz) and equipped with a Bruker 7 mm double-channel probe, and samples were spun at a spinning frequency of 4 kHz. Single-pulse magic angle spinning (SPE–MAS) methods were performed using a pulse angle of π/6 (2.1 μs) and a recycling delay of 80 s under high-power proton decoupling conditions (63 MHz). The quantitative determination of the proportions of the different Q^n^ Si species was ensured by the recording conditions [44], and chemical shifts were recorded relative to tetramethylsilane. Decompositions of the spectra were performed using the Dmfit software (2002, CEMHTI, Paris, France) [45].

### 2.3. VOC Adsorption Measurements

#### 2.3.1. Experimental Measurements

A manometric method was used to assess the sorption capacity of ZK-4 powder (with Si/Al molar ratio of 2.1) and ZK-4 microspheres synthesized at 180 °C for 24 h towards *n*-hexane. For this purpose, a Micromeritics ASAP 2020 device (Norcross, GA, USA) fitted with a vapor generator was used. Prior to each manometric measure, the zeolitic samples were outgassed to a residual pressure of less than 0.8 Pa at 300 °C for 15 h to remove all adsorbate traces. The characteristics of the *n*-hexane are summarized in Table 1. Experiments were performed on 50 mg of zeosil.

#### 2.3.2. CB–GCMC Simulation

We have performed configurational bias grand canonical Monte Carlo (CB–GCMC) simulations of the adsorption of *n*-hexane within the ZK-4 zeolite in order to corroborate the experimental data. The CB–GCMC algorithm implemented in the code “Towhee” was used to achieve this task. The details of our simulation approach as well as the parameters of the simulations are provided as supplementary information.

## 3. Results and Discussion

### 3.1. XRD Analysis

**Figure 1:**
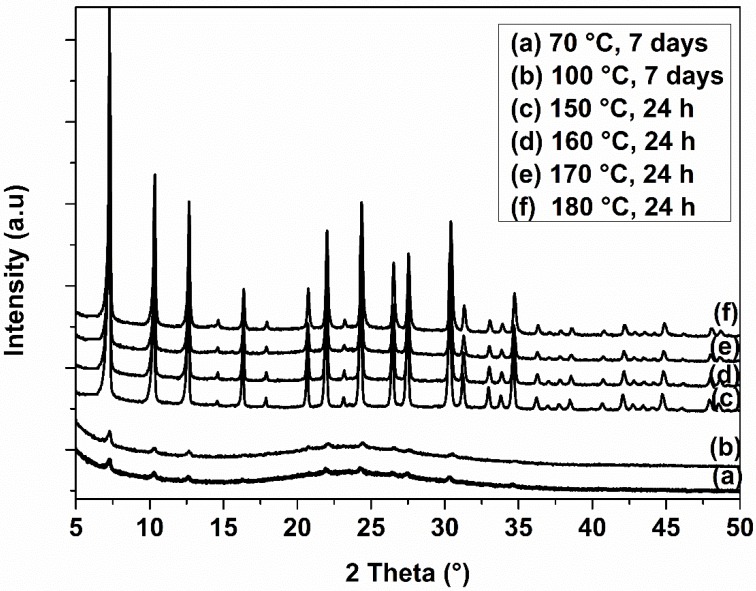
XRD patterns of ZK-4 microspheres synthesized at different temperatures and treatment times.

XRD patterns of the six calcined samples synthesized at different temperatures with duration of hydrothermal treatment of 24 h and 7 days are shown in Figure 1. The 7-days-treated samples prepared at temperatures of 70 °C and 100 °C were in the majority amorphous. Some few wide peaks (due to the small particle size as measured by SEM imaging, Figure 4) of very low intensity attributed to ZK-4 zeolite were detected in these samples. Higher temperatures (ranging from 150 °C to 180 °C) and shorter treatment time (24 h) resulted in a pure ZK-4 crystalline phase with enhanced crystallization rate and particle size (Figure 4). This was reflected by more intense and sharper ZK-4 characteristic peaks and indicates that the crystallinity of the spheres depends on the temperature and treatment time of the synthesis process. 

**Figure 2:**
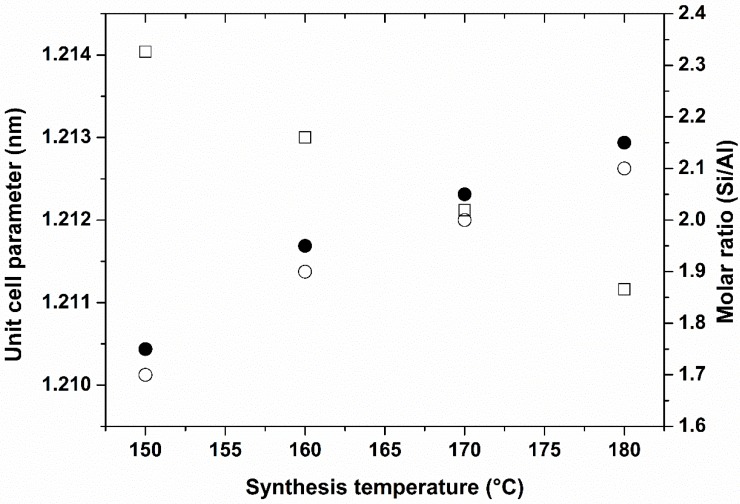
Evolution of the Si/Al molar ratio (full circles determined from XRF analysis and empty circles determined from ^29^Si solid-state NMR) and the unit cell parameter (empty square; value determined from XRD refinement) of the ZK-4 zeolite framework depending on hydrothermal treatment temperature after 24 h.

The unit cell parameter of the four well-crystallized samples obtained from the refinement of the XRD patterns using Win X Pow software decreases with the increase of treatment temperature (Figure 2). This phenomenon can be attributed to the increase of the Si/Al molar ratio of the ZK-4 framework (some Al atoms are replaced by Si atoms in the framework) [47]. This hypothesis was confirmed by the results obtained by XRF and solid-state NMR spectroscopy analyses which show an increase of Si/Al molar ratio of the samples as the treatment temperature increases (Figure 2). This result is very interesting because the Si/Al ratio of the zeolite framework is known to control the catalytic properties of the zeolite and its thermal stability. The thermal stability of the zeolite framework increases with increased Si/Al molar ratio of the framework [48].

**Figure 3:**
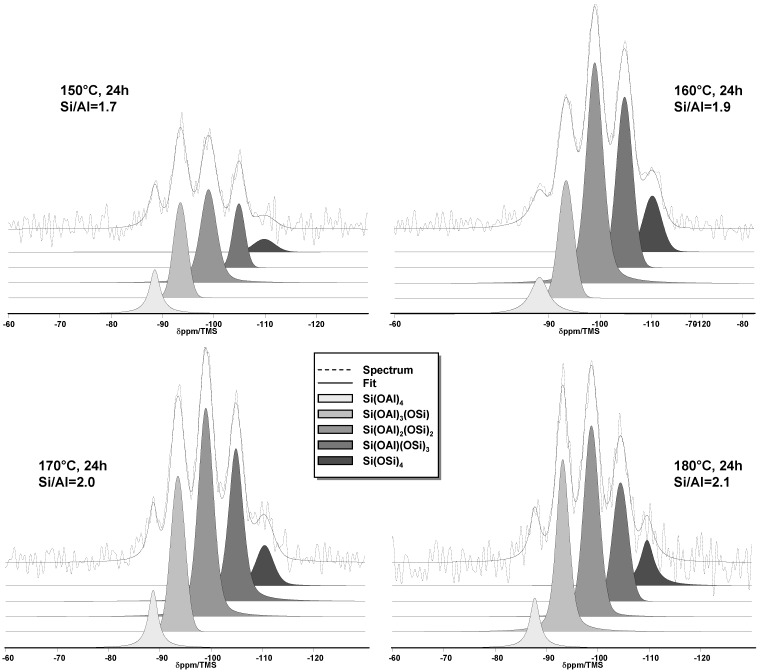
^29^Si MAS + DEC NMR (Magic Angle spinning spectra with heteronuclear DECoupling) spectra of the calcined samples after a hydrothermal treatment of 24 h at 150 °C, 160 °C, 170 °C and 180 °C.

Five ^29^Si MAS NMR resonances can be observed in the spectra (Figure 3) of the four calcined materials obtained after a hydrothermal treatment of 24 h at 150 °C, 160 °C, 170 °C and 180 °C. These five ^29^Si signals are all assigned to Q_4_ units. The resonances observed at approximately −88 ppm, −93 ppm, −99 ppm, −105 ppm and −110 ppm can be assigned to (Si(OAl)_4_), (Si^-^(OSi)_1_(OAl)_3_), (Si^−^ (OSi)_2_(OAl)_2_), (Si(OSi)_3_(OAl)_1_) and (Si(OSi)_4_), respectively [49]. Si/Al ratios were calculated by applying the formula:

(1) $$(\mathrm{Si}/\mathrm{Al}{)}_{\mathrm{NMR}}=\frac{I_{4}\;+\;I_{3}\;+\;I_{2}\;+\;I_{1}}{I_{4}\;+\;0.75I_{3\;}+\;0.5I_{2}\;+\;0.25I_{1}},\;$$

where *I*_n_ is the area of the NMR peak corresponding to the Si(nAl) building unit. The calculated Si/Al ratios obtained for the four samples are consistent with the results from XRF analysis.

**Figure 4:**
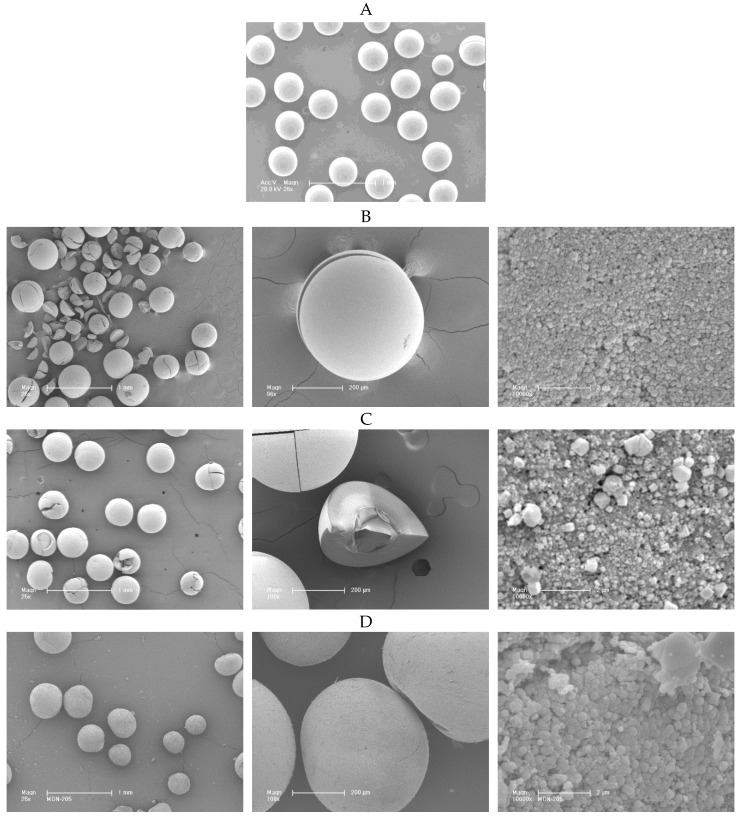

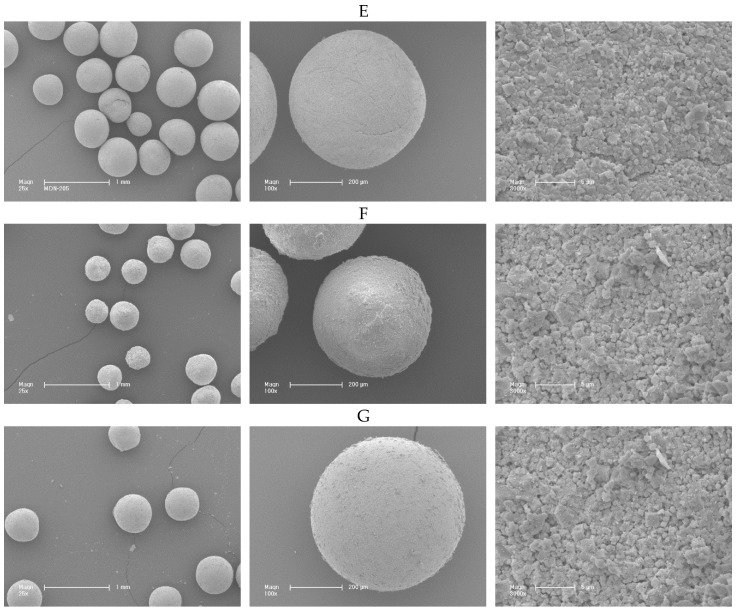
SEM images of MSA-1 resin (**A**) and ZK-4 beads synthesized at different temperatures and different treatment times: (**B**) 70 °C, 7 days, (**C**) 100 °C, 7 days, (**D**) 150 °C, 24 h, (**E**) 160 °C, 24 h, (**F**) 170 °C, 24 h and (**G**) 180 °C, 24 h.

SEM images of the used MSA-1 resin and the zeolite beads obtained at the different temperatures are shown in Figure 4A–G. At the lowest temperatures (70 and 100 °C) and for a treatment duration of 7 days, the microspheres were cracked and even broken (Figure 4B,C), probably due to the high contact time of MSA-1 resin in basic pH of the starting mixture (7 days) which increased the probability of dissolution. Decreasing the contact time from 7 days to 24 h and increasing the hydrothermal treatment temperature allowed us to obtain zeolite beads without cracks and with increased crystallinity. In general, no shrinkage or change in appearance were observed upon removal of the ion exchanger at temperatures starting at 150 °C and for a treatment duration of 24 h (Figure 2D–G). Under these conditions, ZK-4 crystals presented a rough surface which was marked in the case of the microspheres synthesized at 170 and 180 °C. Synthesized microspheres showed diameters between 300 and 600 µm, similar in size to the original resin beads (see Figure 2A). The particle size of the beads increased with the increase of the treatment temperature: below 100 nm, 100–200 nm, 200–360 nm, 550–750 nm, 650–1000 nm and 700–1200 nm for the samples synthesized at 70 °C, 100 °C, 150 °C, 160 °C, 170 °C and 180 °C, respectively. This phenomenon was already observed in previous studies [48,50]. The results demonstrate the effect of temperature and reaction time on the crystallization rate, surface texture and size of the ZK-4 particles constituting the beads. 

**Figure 5:**
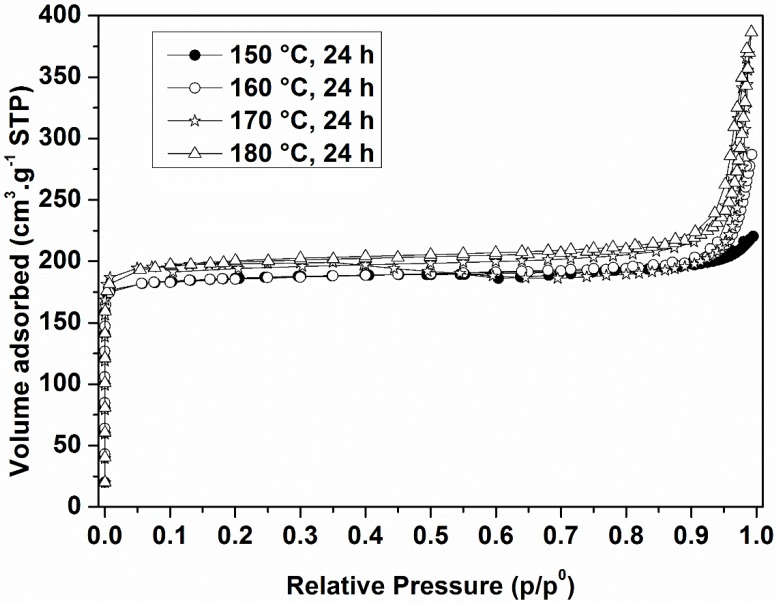
N_2_ adsorption isotherms of the calcined samples after a hydrothermal treatment of 24 h at 150 °C, 160 °C, 170 °C and 180 °C.

The changes in the pore structure of synthesized macrostructures under 24 h treatment duration were studied by nitrogen adsorption. The nitrogen adsorption isotherms recorded for all spheres were of type IV with a steep increase at low relative pressures indicating a substantial microporosity (type I) (Figure 5). The isotherms are representative of zeolitization within the resin which resulted in a material with specific macromorphology and combination of micro- and mesopores. Formed mesopores are related to the removal of the ion exchanger, whereas the micropores are due to the presence of the zeolite ZK-4 building up the structure of the spheres. Accordingly, besides the self-bonded form of the macrostructures, another achieved advantage of the resin templating method is the controlled dual-pore structure of the spheres. This can be explained by the removal of the resins by calcination whereby a solid ZK-4 skeleton is formed in the location of the former interstitial spaces, and the interconnected mesopores occupy the original sites of the resin polymer chains. 

BET surface areas and microporous and mesoporous volumes are listed in Table 2. In general, an increase in the BET surface area and mesopore volume is observed with increased treatment temperatures. An exception is the observed decrease in the BET surface areas of the zeolite ZK-4 spheres synthesized at 180 °C as compared to the samples synthesized at 170 °C, probably due to the increase of particle size. On the other hand, total pore volumes representing both microporous and mesoporous volumes were higher. Similar microporous volumes were measured for the different ZK-4 zeolites. These values are the highest microporous volumes that can be observed for ZK-4 zeolite (LTA-type zeolite), which means that these four zeolite samples are well crystallized, as it was shown from XRD results.

The ZK-4 spheres synthesized at 180 °C present the highest total pore volume, which makes them the ideal candidates for VOC adsorption. This result indicates that under the tested experimental conditions, the use of higher temperature favors the formation of a high porous volume of the material. The BJH pore size distributions showed that in all cases, the majority of the mesopores ranged in size from 40 to 50 nm (figures not shown in the paper). 

### 3.2. n-Hexane Adsorption Analysis

**Figure 6:**
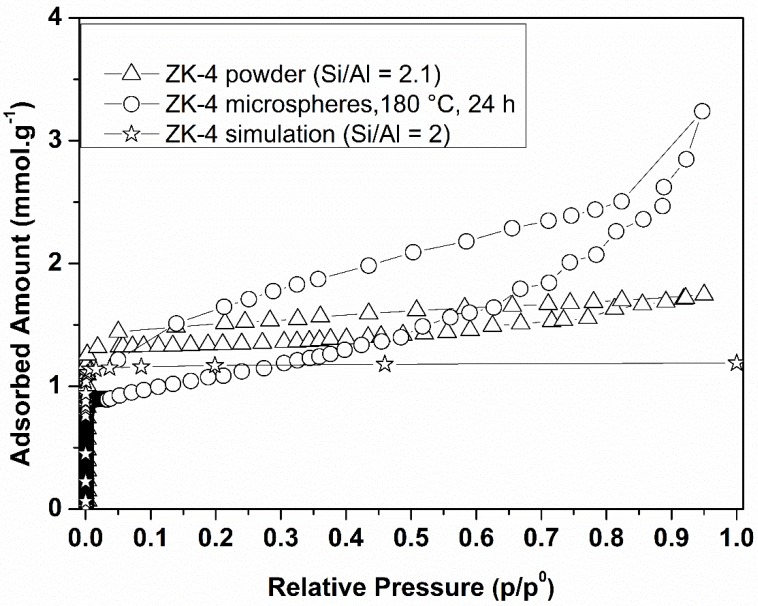
*n*-Hexane adsorption isotherms done at 25 °C.

**Figure 7:**
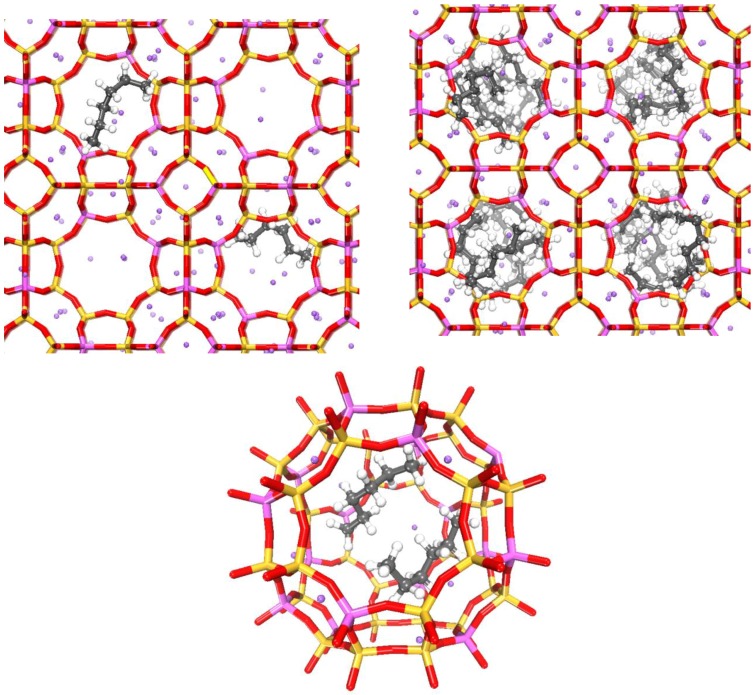
CB–GCMC simulation extracted snapshots illustrating the *n*-hexane molecules’ confinement within the ZK-4 zeolite porosity, respectively at low (on the left) and high (on the right) coverage at 25 °C. The bottom picture shows an arrangement of two *n*-hexane molecules adsorbed within the α-cage.

The adsorption capacity of ZK-4 powder and ZK-4 microspheres synthesized at 150 °C and at 180 °C for 24 h was assessed using *n*-hexane as probe molecule (Figure 6). The ZK-4 zeolite theoretical adsorption capacity was predicted by CB–GCMC simulation (Figure 6), and showed that, at maximum coverage, the ZK-4 zeolite can host two *n*-hexane molecules per unit cell. Only α-cages are accessible to the *n*-hexane molecules; as represented in Figure 7 (bottom), each α-cage is occupied per two *n*-hexane molecules, adopting a mostly parallel arrangement. The zeolite ZK4 powder showed a moderate adsorption capacity of *n*-hexane equal to ~1.40 mmol·g^−1^, which is slightly higher than the one expected by simulation (~1.23 mmol·g^−1^) due to the presence of slight mesoporosity in the ZK-4 powder sample (smashed ZK-4 microspheres). A higher adsorption capacity was measured in the case of ZK-4 microspheres synthesized at 180 °C for 24 h due to the increase in the total pore volume. The adsorption capacity of *n*-hexane using ZK-4 microspheres synthesized at 180 °C reached a significant amount of 3.0 mmol·g^−1^, which is twice as high as the one observed for ZK-4 zeolite powder. The enhanced adsorption capacity of the microspheres is associated with the introduction of mesoporosity in the shaped samples (zeolite microspheres). It is also noteworthy to mention the high desorption capacity (by reducing the relative pressure at 25 °C) of the ZK-4 microspheres as shown in Figure 6, allowing retrieval of the loaded physisorbed pollutant from the adsorbent and the reuse of the zeolite microspheres for molecular decontamination. This avoids the secondary pollution by the disposal of pollutant-loaded adsorbents. 

## 4. Conclusions

ZK-4 microspheres were prepared by one-shot synthesis using macroporous strongly basic ion-exchange resins as shape-directing agents. Besides the generation of a desired macromorphology, resins allowed the synthesis of materials with multilevel porosity. Crystallinity, pore volume and particle size of the ZK-4 microspheres were dependent on the treatment time and temperature of the synthesis solution. They increased with increasing temperatures for a synthesis duration of 24 h. ZK-4 microspheres synthesized at 180 °C for 24 h achieved the highest adsorption of *n*-hexane. 

## Figures and Tables

**Table 1:** Characteristics of the probe molecule used for the adsorption measurements.

	Boiling Point (°C)	Vapor Pressure at 25 °C ^a^ (KPa)	Molar Volume at 25 °C (L·mol^−1^)	Kinetic Diameter ^b^ (Å)
*n*-Hexane	69	≈ 20.0	0.13198	4.3

^a^ Calculated using the Antoine equation logP = A − B/(T + C); the coefficients A, B and C were found on the NIST experimental database website [46], P is pressure (Pa) and T the temperature (°C). ^b^ The kinetic diameters of the probe molecules were determined using Cerius program.

**Table 2:** Specific surface area, microporous and mesoporous volumes of the four synthesized zeolite ZK-4 microspheres.

	150 °C, 24 h	160 °C, 24 h	170 °C, 24 h	180 °C, 24 h
S_BET_ (m²·g^−1^)	766	773	805	795
V_micro_ (cm^3^·g^−1^)	0.28	0.28	0.29	0.29
V_meso_ (cm^3^·g^−1^)	0.09	0.16	0.29	0.31
Mesoporous diameter (nm)	40–50	40–50	40–50	40–50

## Supplementary Materials

The following are available online.
