# Supplementary material for: Synthesis of Binderless ZK-4 Zeolite Microspheres at High Temperature
Source: Molecules. 2018 Oct 16;23(10):2647. doi: 10.3390/molecules23102647 (PMC6222458; doi:10.3390/molecules23102647)
Supplement: Supplementary file 1 [file molecules-23-02647-s001.pdf]

Supporting Information

Synthesis of Binderless ZK-4 Zeolite Microspheres at High Temperature

Elyssa G. Fawaz [a,b,c], Darine A. Salam [c], Habiba Nouali [a,b], Irena Deroche [a,b], Severinne Rigolet [a,b], Benedicte Lebeau [a,b], T. Jean Daou [a,b,*]

[a] *Université de Haute Alsace (UHA), CNRS, Axe Matériaux à Porosité Contrôlée (MPC), Institut de Science des Matériaux de Mulhouse (IS2M), UMR 7361, 3 bis rue Alfred Werner, F-68093 Mulhouse, France.*

[b] *Université de Strasbourg (UniStra), Strasbourg, France.*

[c] *Department of Civil and Environmental Engineering, Faculty of Engineering and Architecture, American University of Beirut, P.O.Box: 11-0236, Riad El Solh. Beirut, Lebanon*

Computational details

1. Microscopic models of adsorbate, adsorbent and their interaction

1.1 Microscopic model for the adsorbate molecules

The n-hexane molecule was described via the as-called "united atom" model that has already succeeded to reproduce the adsorption behaviour of chain alkanes in various microporous materials such as zeolites [1,2] and/or MOFs [3]. In this model, each $-CH_x-$ group is treated as a single interaction site. Such "united atoms" are then connected by bonds, maintained at fixed distances. The intramolecular interactions are described using a sum of a harmonic angle bending term, with a dihedral angle term, expressed by a cosine series potential. The parameters corresponding to those terms have been taken from the Transferable Potential for the Phase Equilibrium (TraPPE) force field [4] and are summarized in Table S1. The intermolecular interactions between the sorbate molecules were modelled using a sum of repulsion-dispersion potential term expressed via the Lennard-Jones interaction as described in Equation 1 [4]:

$$U_{ij}^{Inter} = 4\varepsilon_{ij}\left[\left(\frac{\sigma_{ij}}{r_{ij}}\right)^{12} - \left(\frac{\sigma_{ij}}{r_{ij}}\right)^{6}\right] \qquad \text{(Equation 1)}$$

In Equation 1 r_{ij} is the distance separating two force centres and ε_{ij} and σ_{ij} are the corresponding LJ interatomic potential parameters. The cross LJ terms were calculated applying the Lorentz-Berthelot combination rule. The bond distances and the interatomic potential parameters for all investigated molecules are summarized in Table S1.

1.2 Microscopic model for the ZK-4 zeolite structure and Interction Potential

The **LTA** zeolite has a cubic unit cell with lattice parameter of a = b = c = 11.92 Å. In order to reproduce the experimental Si:Al ratio of ~2, the zeolite unit cell chemical formula was $Si_{16}Al_8Na_8O_{48}$. The Al atoms were

distributed along the unit cell in order to strictly respect the Löwenstein's Al–O–Al avoidance rule [5] and the Na^+ extra-framework cations were placed in accordance with the experimentally determined crystallographic positions. The zeolite system was assumed to be semi-ionic with partial charges taken from Cygan *et al.* [6], summarized together with the Lennard-Jones parameters in the Table S1.

Table S1. Force field parameters for the ZK-4 zeolite and the n-hexane molecule

**Parameters for the non-bonded interactions**			
**Pseudo-atom**	**ε/k_B (K)**	**σ (Å)**	**q(e)**
**$CH_3(sp^3)$**	98	3.75	0
**$CH_2(sp^3)$**	46	3.95	0
**O (zeolite)**	78.20	3.17	-1.20
**Si (zeolite)**	0.93E-3	3.30	2.40
**Al (zeolite)**	0.93E-3	3.30	1.40
**Na^+ (zeolite)**	65.47	2.35	1.00
**Parameters for the bond potential**			
**Bond lengths (rigid bond)**			
**Type**		**r_0 (Å)**	
**CH_3-CH_2**		1.54	
**CH_2-CH_2**		1.54	
**Bending potential parameters**<br>**$U_{bending} = k_\theta (\cos\theta - \cos\theta_{eq})$**			
**Type**	**θ_{eq}(°)**	**k_θ/k_B (K)**	
**CH_3-CH_2-CH_2**	114.0	31250	
**Dihedral Torsion potential parameters**			

$U_{torsion} = C_0 + C_1 (1+\cos\theta) + C_2 (1-\cos2\theta) + C_3 (1+\cos3\theta)$				
**Type**	**C_0/k_B (K)**	**C_1/k_B (K)**	**C_2/k_B (K)**	**C_3/k_B (K)**
**CH_3-CH_2-CH_2-CH_2**	0.0	355.03	-68.19	791.32
**CH_2-CH_2-CH_2-CH_2**	0.0	355.03	-68.19	791.32

2. CB-GCMC simulation

Absolute adsorption isotherm of *n*-hexane in ZK-4 zeolite at 298K was calculated applying Monte Carlo simulation in the Grand Canonical ensemble (μVT) [7], as implemented within the code Towhee [8]. This simulation method allows evaluating the average number of adsorbed molecules in equilibrium with an infinite reservoir of molecules imposing its chemical potential and temperature. The chemical potential values were calculated by the test particle Widom insertion method from the NpT ensemble Monte Carlo simulation [9]. It is now well known that the conventional GCMC simulation scheme is inefficient for flexible, long chain molecules because of the extremely low fraction of successful insertion moves. In order to achieve a higher acceptance probability, we applied the configurational-biased algorithm, which selects an energetically favourable phase space for insert the adsorbate [10]. The simulation box consisted of 27 unit cells (unit cell multiplied by 3 in x, y and z space directions). The framework atoms (Si, Al and O) were maintained fixed during the simulation, whereas all Na^+ compensating cations were allowed to relax during the MC run. The periodic conditions were applied in all directions of the space. A typical Monte Carlo run consisted of 3.0×10^7 steps for the equilibration phase and the same number of steps for the production phase. Each step corresponded to a single MC move, including a centre of mass translation, centre of mass rotation, insertion of a new molecule, deletion of a randomly selected existing molecule, partial or complete regrowth of the sorbate. Cation positions relaxation was achieved by translation movement. Finally, the Ewald summation method was applied in order to calculate the electrostatic part of the interaction energy and the short-range interactions were truncated at a cut-off distance of 14.0 Å.

References

[1] Pascual P.; Ungerer P.; Tavitian B.; Pernot P.; & Boutin A. Development of a transferable guest–host force field for adsorption of hydrocarbons in zeolites : I. Reinvestigation of alkane adsorption in silicalite by grand canonical Monte Carlo simulation. **2003**, *Phys. Chem. Chem. Phys.*, *5*(17), 3684–3693. https://doi.org/10.1039/B304209J.

[2] Cosseron A.F.; Daou T. J.; Tzanis L.; Nouali H.; Deroche I.; Coasne B.; & Tchamber V. Adsorption of volatile organic compounds in pure silica CHA, ∗BEA, MFI and STT-type zeolites. **2013**, *Microporous and Mesoporous Mater.*, *173*, 147–154. https://doi.org/10.1016/j.micromeso.2013.02.009.

[3] Déroche I.; Rives S.; Trung T.; Yang Q.; Ghoufi A.; Ramsahye N. A.; … Maurin G. Exploration of the Long-Chain N -Alkanes Adsorption and Diffusion in the MOF-Type MIL-47 (V) Material by Combining Experimental and Molecular Simulation Tools. **2011**, *J. Phys. Chem. C*, *115*(28), 13868–13876. https://doi.org/10.1021/jp2039527.

[4] Martin, M.G.; Siepmann, J.I. Transferable Potentials for Phase Equilibria. 1. United-Atom Description of *n*-Alkanes. **1998**, *J. Phys. Chem. B*, 102, 2569–2577. https://doi.org/ 10.1021/jp972543+.

[5] Klinowski J. Applications of solid-state NMR for the study of molecular sieves. **1993**, *Analyt. Chim. Acta*, *283*(3), 929–965. https://doi.org/10.1016/0003-2670(93)80258-M.

[6] Cygan R. T.; Liang J.J.; & Kalinichev A. G. Molecular Models of Hydroxide, Oxyhydroxide, and Clay Phases and the Development of a General Force Field. **2004**, *J. Phys. Chem. B*, *108*(4), 1255–1266. https://doi.org/10.1021/jp0363287.

[7] Frenkel, D.; Smit, B. Understanding Molecular Simulations: From algorithms to applications, Academic Press: New York, **1996**.

[8] Martin M. G. MCCCS Towhee: a tool for Monte Carlo molecular simulation. **2013**, *Molecul. Simul.*, *39*(14–15), 1212–1222. https://doi.org/10.1080/08927022.2013.828208.

[9] Widom B. Some Topics in the Theory of Fluids. **1963**, *J. Chem. Phys.*, *39*(11), 2808–2812. https://doi.org/10.1063/1.1734110.

[10] Siepmann J. I.; & Frenkel D. Configurational bias Monte Carlo: a new sampling scheme for flexible chains. **1992**, *Molecul. Phys.*, *75*(1), 59–70. https://doi.org/10.1080/00268979200100061.
